# Prevalence and risk factors for low back pain during pregnancy among women in Abakaliki, Nigeria

**DOI:** 10.11604/pamj.2021.39.70.24367

**Published:** 2021-05-26

**Authors:** Njoku Isaac Omoke, Peace Ifeoma Amaraegbulam, Odidika Ugochukwu Joannes Umeora, Love Chimezirim Okafor

**Affiliations:** 1 Department of Surgery, Ebonyi State University/Alex Ekwueme Federal University Teaching Hospital, Abakaliki, Nigeria,; 2 Department of Surgery, Federal Medical Centre, Umuahia, Nigeria,; 3 Department of Obstetrics and Gynaecology, Ebonyi State University/Alex Ekwueme Federal University Teaching Hospital, Abakaliki, Nigeria,; 4 Department of Obstetrics and Gynaecology, Alex Ekwueme Federal University Teaching Hospital, Abakaliki, Nigeria

**Keywords:** Prevalence, low back pain, pregnancy women, risk factors, Nigeria

## Abstract

**Introduction:**

low back pain (LBP) during pregnancy is an important health concern among women globally. The prevalence and risk factors for LBP in pregnancy vary from and within sub-regions and have implications in preventive and treatment strategies. In West African sub-region, there is scanty data on LBP during pregnancy. This study aimed to determine the prevalence and predisposing factors for LBP during pregnancy in this environment.

**Methods:**

this was a cross-sectional study carried out among pregnant women admitted into the Labour Ward of Alex Ekwueme Federal University Teaching Hospital, Abakaliki, Nigeria over a period of 8 months. They were interviewed within 2 to 7 days postpartum with a questionnaire. Sociodemographic and obstetrics characteristics as well as LBP intensity, features and associated factors were evaluated. Significant factors for LBP that emerged from the univariable analysis were entered into multivariable regression analysis to evaluate the risk of each factor when adjusted to other factors.

**Results:**

of the 478 women interviewed, 138 (28.9%) of them (95% CI 25.1-33.1) reported LBP in the index pregnancy. The onset of pain was predominantly in the third trimester and the mean pain intensity was 4.3 ± 1.36. In the univariable analysis, six factors were significantly associated with LBP. Logistic regression analysis identified LBP in previous pregnancy (aOR: 24.76, (95% CI 6.88-89.11); p< 0.001), macrosomia (aOR: 4.15(95% CI 2.05-8.42); p< 0.001) and absence of domestic help (aOR: 0.50(95% CI 0.31-0.82); p=0.006) as independent risk factors for LBP during pregnancy among the women.

**Conclusion:**

in this study, LBP during pregnancy is within worldwide range and predominantly mild to moderate in intensity. The independent risk factors identified call for high priorities accorded to women with these factors in measures aimed at addressing LBP during pregnancy.

## Introduction

Low back pain (LBP) is a common health concern among women during pregnancy worldwide. The prevalence of low back pain during pregnancy varies from and within sub regions; it ranges from 24-90% [1-10]. Moderate to severe disability associated with low back pain is often a burden in pregnancy [11]. The negative impact of low back pain during pregnancy has implications on maternal quality of life and satisfaction with pregnancy [7-12]. A recently published report indicates that the duration of low back pain in pregnancy correlates directly with the duration of absenteeism, physical dysfunction and poor work performance [2]. Quite saddening is the fact that many pregnant women with low back pain do not complain to their health caregivers, while those who complain often have their complaints ignored [6,7]. Therefore, it is imperative that the antenatal caregivers are well armed with clinical skills to recognise and deal with low back pain during pregnancy early for a better outcome [6].

Pregnancy-related low back pain is any type of idiopathic pain arising between the lower margin of the 12^th^ rib and the inferior gluteal folds during the course of pregnancy [7,12]. The aetiology is poorly understood but is often ascribed to mechanical, hormonal or a combination of factors associated with the body changes in pregnancy [12,13]. The risk factors for LBP in pregnancy vary in published reports and there is no consensus about the predisposing factors [7,8,12-14]. However, chronic low back pain and LBP in a previous pregnancy are the most commonly identified risk factors in published reports [8,12,14]. The severity and impact of LBP during pregnancy also differ across countries; in published reports the mean pain intensity ranges from 3.7 to 7 on the pain Numeric Rating Scale (NRS) [5,6,8]. Additionally, Gutke *et al*. in a multinational study that included women population in the United Kingdom (UK), United State of America (USA), Norway and Sweden demonstrated that UK women reported the highest pain intensity and impact of LBP in pregnancy whereas USA women despite the highest prevalence of LBP reported were the least afflicted [6]. Thus, a good knowledge of LBP during pregnancy and its predisposing factors in a setting is important and can facilitate preventive strategies and tailored interventions to ensure optimum care.

However, in the West African sub-region, there is very scanty data on LBP during pregnancy. The few previously published reports from the sub-region focused more or less on the prevalence and pattern of back pain during pregnancy [2,10,15,16]. This implies that data on risk factors for LBP during pregnancy in the sub region is almost non-existent. The variations in the prevalence and risk factors for LBP during pregnancy in previously published reports underscore the importance of data from this environment. Therefore, this study aimed to determine the prevalence and risk factors for LBP during pregnancy among women in a South Eastern Nigerian setting.

## Methods

**Study setting and design:** this study was a cross-sectional questionnaire-based survey carried out among pregnant women admitted into the labour ward of Alex Ekwueme Federal University Teaching Hospital Abakaliki, Ebonyi State, Nigeria (formerly, Federal Teaching Hospital Abakaliki). The hospital came into existence in the year 2011 after a successful merger of the Ebonyi State University Teaching Hospital and Federal Medical Centre Abakaliki by the Federal Government of Nigeria, and is one of the major teaching hospitals South-East Nigeria.

**Ethical approval:** the approval for this study was obtained from the Ethics and Research Committee of Alex Ekwueme Federal Teaching Hospital Abakaliki, Nigeria (Fetha/REC/vol.1 2014/175). A written informed consent was obtained from the patient before the study instruments were administered.

**Inclusion criteria:** all clinically stable and consenting pregnant women admitted into the obstetric ward and underwent delivery during the confinement.

**Exclusion criteria:** women who objected to giving informed consent, history of spinal or rheumatologic disorder, history of vertebral spine fracture or surgery, previous significant lumbar magnetic resonance imaging (MRI) finding, women with cognitive impairment, and chronic pain syndromes.

**Instrument of study:** a questionnaire designed by the research group captured the biodata of the participants as well as social habits of the participant and her husband (alcohol and tobacco consumption), history of domestic violence, presence/absence of domestic help, and pre-existing medical conditions. Variables relating to LBP obtained included: presence/absence of pain in index pregnancy, the gestational age (GA) at the first episode of pain in index pregnancy, the severity of pain (using Numeric Rating Scale (NRS), where 0 represented no pain and 10 highest intensity of pain), aggravating factors for LBP, history of LBP in a previous pregnancy, health seeking behaviour (complaint to doctor/caregiver) and treatment received for the LBP. Information obtained concerning obstetric history included: parity and gravidity, history of previous spontaneous abortion (the GA it occurred), history of dysmenorrhoea, booking status of index pregnancy, single/multiple gestations, mode of delivery of baby in index pregnancy and birth weight of the product of index pregnancy.

**Procedure:** four hundred and seventy-eight (478) consecutive and consenting pregnant women who met the inclusion criteria were recruited into the study from March 2019 to October 2019 in the obstetric ward. They were interviewed with the questionnaire, while on admission in the ward, 2 to 7 days after delivery. The literate ones filled the questionnaire in English while one of the investigators and a trained research assistant who understand the local dialect helped the non-literate women. The patients were classified based on employment status into unemployed, employed and students for analysis. In this study, the employed includes the self-employed patients (farmers, artisans, traders etc.) government and private sector employees. The birth weight of the product of index pregnancy was considered as a probable risk factor for LBP and the patients were classified into two groups based on it (baby birth weight <4Kg vs ≥4Kg, macrosomia) for analysis.

**Statistical analysis:** data analysis was carried out using Statistical Package for Social sciences SPSS version 20 (SPSS Inc; Chicago, Illinois., univariable analysis was carried out and p value set at <0.20 for statistically significant factors. The statistically significant factors that emerged from the univariable analysis were entered into a stepwise logistic regression model for multivariable analysis to evaluate the risk of each factor when adjusted to other factors. In the multivariable regression analysis, the p value for the predictors of low back pain in pregnancy was set at p <0.05.

## Results

**Prevalence of low back pain:** there were 478 consecutive and consenting women that were recruited and interviewed in this study and 138 (28.9%) of them (95% CI 25.1-33.1) reported having LBP during the index pregnancy. Of the 138 women that reported LBP in the index pregnancy, the majority (119, 86.2%) of them experienced LBP during pregnancy for the first time whereas (19, 13.8%) of them reported episodes of low back pain in a previous pregnancy. Of the 119 that had the first episode of LBP was in the index pregnancy, 65 (54.6%) and 54 (45.4%) of them were Multipara and Primipara women respectively. Of the 19 that had LBP in previous pregnancy, 17 (89.5%) and 2 (10.5%) were multipara and primipara women respectively.

**Pattern of low back pain:** of the 138 with low back pain in index pregnancy, the mean pain intensity on NRS was 4.3±1.36, and the pain was mild, moderate and severe in 42 (30.4%), 87 (63.0%) and 9 (6.5%) of them respectively. The pain occurred in the first trimester (3, 2.2%), second trimester (54, 39.1%) and third trimester (81, 58.7%) of pregnancy. Of the 138 that reported LBP in index pregnancy, the aggravating factor for pain was postural (51, 37%), strenuous physical work (33, 23.9%), postural and strenuous work (4, 2.9%) whereas there was no aggravating factor in 50 (36.2%) of the respondent. Eighty-one (58.7%) informed the antenatal caregiver about the low back pain whereas 57 (41.3%) did not complain about it. Of the 81 patients that complained to the caregiver, 38 (46.9%) were given words of assurance, 26 (32.1%) were treated with analgesics and 17 (21%) reported that the caregiver ignored the complaint of LBP.

**Sociodemographic and obstetric characteristics of the population:**Table 1 shows the statistical description of sociodemographic variables of the population that were considered for inclusion in the analysis. The mean age of the respondents was 29.33 ± 4.8 years. There was no significant difference in the mean age (29.23 ± 4.29 years) of the women with LBP and the mean age (29.38 ± 4.4.9 year) of the women that had no LBP (p=0.178]. The incidence of LBP was highest among the 41 - 45-year-old group, unemployed women and women without formal education as also shown in Table 1. The incidence of low back pain during pregnancy was higher among the Multiparous women, in those with previous history of low back pain during pregnancy and index baby weight >4Kg as shown in Table 2. Table 3 shows domestic and lifestyle variables included in the analysis. Also, in Table 3, the incidence of LBP during pregnancy was higher in the absence of domestic help compared to the presence of domestic help during pregnancy.

**Table 1:** low back pain during pregnancy by sociodemographic characteristic of the participants

Demographic variables	Low back pain in pregnancy		Total (%)
	**NO (%)**	**YES (%)**	
**Age (year)**			
16-20	16 (66.7)	8 (33.3)	24 (5)
21-25	63 (75.0)	21 (25.0)	84 (17.6)
26-30	119 (66.9)	59 (33.1)	178 (37.2)
31-35	109 (74.7)	37 (25.3)	146 (14.6)
36-40	30 (73.2)	11 (26.8)	41 (8.6)
41-45	3 (60.0)	2 (40.0)	5 (1.0)
**Religion**			
Christians	338 (71.3)	136 (28.7)	474 (99.2)
Moslems	2 (50.0)	2 (50.0)	4 (0.8)
**Marital status**			
Married	336 (71.3)	135 (28.7)	471 (98.5)
Single	4 (57.1)	3 (42.9)	7 (1.5)
**Educational status**			
None	0 (0.0)	4 (100.0)	4 (0.8)
Primary	44 (77.2)	13 (22.8)	57 (11.9)
Secondary	133 (72.1)	51 (27.7)	184 (38.5)
Tertiary	163 (70.0)	70 (30.0)	233 (48.7)
**Employment status**			
Unemployed	46 (58.2)	33 (41.8)	79 (16.5)
Employed	243 (73.9)	86 (26.1)	329 (68.8)
Students	51 (72.9)	19 (27.1)	70 (14.6)

**Table 2:** low back pain in pregnancy by obstetrics characteristics

Obstetrics related variables	Low back pain in pregnancy		Total (%)
	**NO (%)**	**YES (%)**	
**Parity**			
Primipara	105 (62.5)	54 (34.0)	159 (33.3)
Multipara	235 (73.7)	84 (26.3)	319 (66.7)
**Booking status**			
Booked	251 (71.1)	102 (28.9)	353 (73.8)
Unbooked	89 (71.2)	36 (28.8)	125 (26.2)
**History of spontaneous abortion**			
Yes	41 (73.2)	15 (26.8)	56 (11.7)
No	299 (70.9)	123 (29.1)	422 (88.3)
**History of dysmenorrheal**			
Yes	12 (66.7)	6 (33.3)	18 (3.8)
No	328 (71.3)	132 (28.7)	460 (96.2)
**Previous low back pain in pregnancy**			
No	337 (73.9)	119 (26.1)	456 (95.4)
Yes	3 (13.6)	19 (84.4)	22 (4.6)
**Multiple gestations index pregnancy**			
Yes	1 (25.0)	3 (75.0)	4 (0.8)
No	339 (71.5)	135 (28.5)	474 (99.2)
**Index baby birth weight(s) (Kg)**			
<4	321 (73.3)	117 (26.7)	438 (91.6)
≥4	19 (47.5)	21 (52.5)	40 (8.4)

**Table 3:** low back pain by domestic and lifestyle characteristics

Domestic and life style variables	Low back pain in pregnancy		Total (%)
	**NO (%)**	**YES (%)**	
**Domestic help**			
Yes	194 (76.4)	60 (23.6)	254 (53.1)
No	146 (65.2)	78 (34.8)	224 (46.9)
**Alcohol consumption**			
Husband	129 (72.2)	38 (22.3)	167 (34.9)
Wife	1 (100)	0 (0.0)	1 (0.2)
Both	1 (16.7)	5 (83.3)	6 (1.3)
None	209 (68.8)	95 (31.3)	304 (63.6)
**Tobacco smoking/snuffing**			
Husband	15 (68.2)	7 (31.8)	22 (4.6)
Wife	1 (100)	0 (0.0)	1 (0.2)
Both	--	--	--
None	324 (71.2)	131(28.8)	455 (95.2)

**Risk factors for low back pain during pregnancy:** in univariable analysis, employment status, parity, history of previous LBP in pregnancy, Index baby weight >4Kg weight, absence of domestic help and alcohol consumption were identified as factors associated with low back pain during pregnancy as shown in Table 4. The result of multivariable logistic regression analysis to determine the risk of each factor when adjusted to other factors was also summarized as shown in Table 4. In the multivariable analysis, previous history of LBP in pregnancy (aOR: 24.76, 95%CI. 6.88-89.11; p< 0.001), baby birth weight of >4kg (aOR: 4.15(2.05-8.42; p < 0.001) and absence of domestic help (aOR: 0.50 (0.31 - 0.82); p=0.006) were identified as independent risk factors for LBP during pregnancy. In Table 4, the odd of LBP in pregnancy was 28.8 times higher in a woman with a history of LBP in previous pregnancy compared to those without LBP in a previous pregnancy. Women carrying unborn macrocosmic baby were 4.2 times more likely to report LBP than those with normal fetal weight. Women that have no domestic help were more likely to report LBP than those that have domestic help.

**Table 4:** univariable and multivariable predictors of low back pain during pregnancy

	Univariable analysis		Multivariable analysis	
**Population characteristics**	**OR (95% CI)**	**p Value**	**AOR (95% CI)**	**p Value**
Age	0.962 (0.794 -1.165)	0.692		
Religion	0.402 (0.056 - 2. 885)	0.365		
Marital Status	0.536 (0.118 - 2.426)	0.418		
Educational status	0.748 (0.349 - 1.357)	0.691		
**Employment status**		0.103		0.115
Employed	1.632 (0.814- 3.269)	0.167	1.999 (.937 - 4.227)	0.073
Student	0.938 (0.523-1.680)	0.829	1.159 (0.609 - 2.206)	0.653
Parity	0.758 (0.524 - 1.096)	0.141	0.731 (0.474 - 1.186)	0.215
Booking Status	1.005 (0.640 - 1.576)	0.984		
History of spontaneous abortion	0.889 (0.475 - 1.660)	0.714		
History of dysmenorrheal	1.242 (0.457 - 3.379)	0.671		
Previous LBP in pregnancy	17.936 (5.214 - 61.697)	0.001	24.758 (6.878 - 89.169)	<0.001
Multi gestation index pregnancy	0.886 (0.408 - 1.922)	0.759		
Index baby weight/s (Kg)	3.032 (1.574 - 5.842)	0.001	4.153 (2.049 - 8.417)	<0.001
Domestic Help	0.579 (0.388 - 0.863)	0.007	0.504 (0.309 - 0.824)	0.006
**Alcohol consumption**		**0.028**		**0.062**
Wife	0.648 (0.419 - 1.002)	0.051	0.635 (0.395- 1.021)	0.061
Both	0.000 (0.000)	1.000	0.000 (0.000)	1.000
None	11.000 (1.268 -095.448)	0.030	8.228 (0.840-80.607)	0.070
Tobacco smoking/ snuffing	1.154 (0.460 - 2.890)	0.954		

OR: odd ratio; CI: confidence interval; AOR: adjusted odd ratio; Multi: multiple

## Discussion

The prevalence of low back pain during pregnancy in this study is within the worldwide range [1-10]. It is close to a prevalence of 33.2% reported from Ethiopia but quite at variance with the prevalence of over 50% in published reports from Akure and Ilorin Nigeria, Malawi, Iran, Turkey and USA [2,4,6-10]. The exact reason for the relatively low prevalence of LBP during pregnancy in this study compared to the rates in most other published reports is not evident. However, pain is subjective and sociocultural circumstances among other factors influence how a woman perceives and cope with low back pain in pregnancy [17]. Perception of pregnancy-related pain such as LBP and labour pain as normal and expected is common among women in the setting of this study, and is a plausible explanation for the relatively low prevalence of LBP observed [18].

The mean pain intensity in this study is close to 4.9 reported by Saxena *et al*. in India but differs from a lower mean pain intensity of 3.7 reported by Sencan *et al*. in Turkey, and a higher mean pain intensity of 7 for UK women reported by Gutke *et al*. [6,8]. The exact reason for the differences in the mean pain intensity observed in this study compared to reports from Turkey and UK is not evident. However, the intensity of pain reported is a reflection of the subjectivity of the pain and sociocultural circumstances that affect coping and perception of LBP during pregnancy. The occurrence of LBP predominantly in the third trimester of pregnancy in this study is also similar to the finding in most published reports [4,9].

In this study, the percentage of the women that reported to the antenatal care giver about the LBP, compared to the findings of Gutke *et al*. in a multinational survey, is similar to 59% for USA women but differs from 66% and 89% for the UK and Norwegian women respectively [6]. Low back pain in pregnancy is perceived as normal and expected in this setting; USA women are less afflicted and concerned about it than UK and Norwegian women. This perhaps is a plausible explanation for the similarity and differences in the rate of reporting LBP to the antenatal caregiver observed. In this setting, of the patient that reported to the caregiver, about half of them were given only words of assurance and one in five of them were ignored. This suggests that some of the health caregivers also view LBP during pregnancy through the same lens of socio-cultural background of a normal occurrence in pregnancy. Pregnancy related LBP, if not identified or accepted as a problem, is more likely to be ignored and not treated [6]. Therefore, it is important that LBP is sought during the evaluation of pregnant women in antenatal clinic and appropriate treatment given to those afflicted.

The aetiology of LBP in pregnancy though yet to be fully elucidated is generally attributed to changes in the body load and mechanics that occur during the carrying of an unborn child and the effect of hormonal changes during pregnancy on musculoskeletal structures of the lower spine and pelvis [12,19]. Low back pain during pregnancy as in LBP of mechanical origin in the general population can recur or transit to chronicity. Mogren IM demonstrated prevalence of recurrence or continuous LBP six-month postpartum among women with LBP during pregnancy and reported a recurrence rate of 36.2% and continuous LBP in 6.9% of them [20]. This implies that the risk of LBP in a subsequent pregnancy is very high once there is a history of LBP in a previous pregnancy and is consistent with LBP in previous pregnancy that has been reported as a common predisposing factor for LBP during pregnancy in most published reports [8,12,14]. Thus, the history of LBP in previous pregnancy identified as an independent risk factor in this study confirms the findings in these previous reports.

In this study, macrosomia emerging as an independent risk factor for LBP during pregnancy is quite an interesting and unprecedented finding. In previously published reports a strong correlation between macrosomia and maternal obesity as well as pregnancy weight gain was demonstrated [21,22]. Maternal weight gain as high BMI in pregnancy is a risk factor for LBP during pregnancy [5,23]. The weight of the fetus and placenta contribute to maternal pregnancy weight gain. This is perhaps an explanation for macrosomia that was identified as an independent risk factor for LBP in pregnancy.

In the sub-region, pregnant women are not often exempted from heavy workload, and are expected to combine occupational/fieldwork with a multitude of household chores [24]. Some of these household chores, such as sweeping, mopping, cleaning, fetching and carrying buckets of water, splitting and cooking with firewood, childcare and so on, ordinarily stress the low back region, and with changes in body load and mechanics during pregnancy can easily precipitate and aggravate LBP. There is no mechanism of domestic work-sharing and the need for one is often obscured by the cultural background of gender roles [24]. Thus, the husband role is shaped and restricted by cultural practices, and domestic chores are seen as demeaning task for men [24]. Consequently, a pregnant woman may resort to the services of domestic help to fill in the gap, and in the absence of a housework assistance she has no alternative other than carry the entire burden of household chores. Thus, it is not surprising in this study that absence of a domestic help was identified as an independent risk factor for LBP during pregnancy. This also confirms the correlation between LBP during pregnancy and the absence of housework assistance reported by Sencan *et al*. in Turkey [8]. The absence of domestic help though under-recognized is a modifiable risk factor for LBP during pregnancy. This calls for an educational programme to enlighten and emphasize the importance of assisting pregnant women with domestic chores as one of the preventive strategies in pregnancy-related LBP.

The limitation of this study is in being a cross-sectional hospital and a single centre based one. The data obtained may not be a representation of the entire pregnant women population. Despite these limitations, the findings from this study are quite strong and can serve as a baseline data for comparison in future studies.

## Conclusion

In this study, about a third of pregnant women experienced low back pain that was predominantly mild to moderate in intensity and most prevalent in the last trimester of pregnancy. There are several factors associated with LBP during pregnancy; the three independent risk factors identified call for domestic help to pregnant women and priorities accorded to those with a history of LBP in previous pregnancy and macrosomia in the measures aimed at addressing LBP during pregnancy.

### What is known about this topic

- LBP during pregnancy is a common and important health concern among women worldwide;
- The aetiology of LBP during pregnancy is not yet fully elucidated;
- In published reports, the predisposing factors vary but history of LBP in previous pregnancy is the most commonly identified risk factor.

### What this study adds

- A third of pregnant women in Abakaliki South-East Nigeria experience low back pain that is predominantly mild to moderate intensity;
- Macrosomia identified as an independent risk factor for low back pain during pregnancy is an unprecedented risk factor;
- Absence of domestic help though under recognized is a modifiable independent risk factor for LBP during pregnancy.
